# Microbiota-metabolites interaction associated with glycemic improvement following a dietary herbal intervention in type 2 diabetes

**DOI:** 10.3389/fnut.2026.1793130

**Published:** 2026-04-13

**Authors:** Bingbing Li, Zeming Ren, Hongchun Li, Mengdi Li, Haili Zhong, Qichen Nie, Jieteng Chen, Renzhao Wu, Ju-Sheng Zheng, Kui Deng, Yuqun Cai

**Affiliations:** 1 School of Medicine, Westlake University, Hangzhou, China; 2 Institute of Basic Medical Sciences, Westlake Institute for Advanced Study, Hangzhou, China; 3 Institute of Basic Medicine, Zhejiang Academy of Traditional Chinese Medicine, Hangzhou, China; 4 Tongde Hospital of Zhejiang Province, Hangzhou, China; 5 Integrated Biomedical Engineering and Health Sciences, McMaster University, Hamilton, ON, Canada; 6 Guangdong Provincial Key Laboratory of Food, Nutrition and Health, Department of Epidemiology, School of Public Health, Sun Yat-sen University, Guangzhou, China; 7 Department of Integrated Traditional Chinese and Western Medicine, Zhejiang Provincial People's Hospital, Hangzhou, China

**Keywords:** dietary intervention, glycemic control, gut microbiome, metabolomics, prospective cohort, type 2 diabetes

## Abstract

**Background:**

Type 2 diabetes (T2D) is a global metabolic disorder characterized by chronic hyperglycemia and disruption of the gut microbiome. Nutritional and microbiota-targeted interventions have emerged as promising strategies for glycemic management, yet longitudinal clinical evidence integrating microbial and metabolic mechanisms remains limited. This study investigated microbiota-metabolites alterations during a standardized dietary herbal intervention (QingYun7, QY7) and explored their relationship with glycemic regulation across both animal study and clinical settings.

**Methods:**

The metabolic and microbial effects of QY7 were first evaluated in diabetic rats through measurements of blood glucose, and gut microbiota composition. Subsequently, a prospective cohort of 385 patients with T2D received QY7, with longitudinal monitoring of fasting, random, and 2-h postprandial glucose, gut microbiota, and serum metabolites across multiple time points. Fecal microbiota transplantation (FMT) from patients before and after intervention into antibiotic-treated mice was performed to evaluate the causal contribution of the gut microbiome to glycemic improvement. Mediation analyses were conducted to delineate potential pathways linking gut microbes, serum metabolites, and glucose outcomes.

**Results:**

In diabetic rats, QY7 administration significantly reduced blood glucose, and restored gut microbial composition. In the clinical cohort, the intervention was associated with rapid and sustained reductions in fasting, random, and postprandial glucose levels, accompanied by consistent remodeling of the gut microbiome and serum metabolite profile. FMT experiments demonstrated that microbiota derived from post-intervention patients conferred improved glycemic responses in recipient mice, supporting a causal role of gut microbiota in metabolic regulation. Serum metabolomic profiling revealed significant alterations, including enrichment of branched-chain amino acid related pathways. Mediation analyses identified key metabolites, phenyllactic acid, 3-methyl-2-oxobutanoic acid, and anandamide, as mediators linking specific bacterial taxa (*Alistipes shahii* and *Limosilactobacillus mucosae)* to fasting and postprandial glucose levels.

**Conclusion:**

This study provides translational evidence that a dietary herbal intervention associated with glycemic improvement in T2D through microbiota-mediated metabolic reprogramming. Gut microbiome alterations induced by the intervention exerted causal effects on blood glucose regulation, with serum metabolites acting as potential key intermediaries. These findings highlight the mechanistic insight in nutrition-based microbiome modulation strategy in T2D.

## Graphical Abstract

**Figure:**
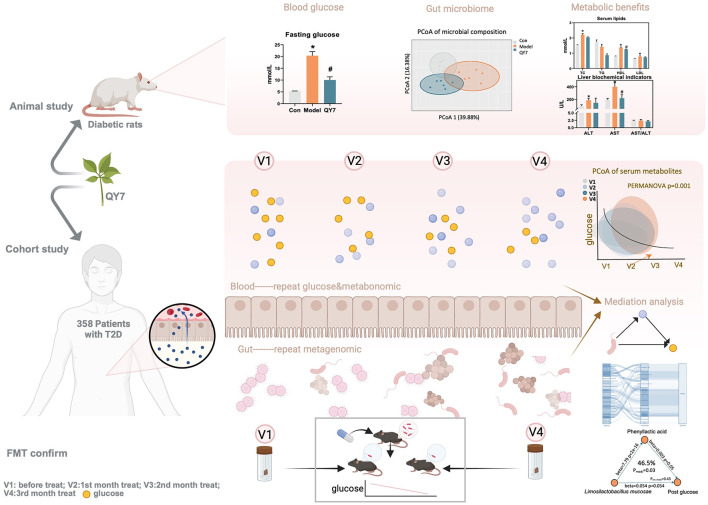

## Introduction

Type 2 diabetes (T2D), a chronic metabolic disorder characterized by insulin resistance and leading to sustained hyperglycemia, accounts for nearly 90% of approximately 537 million cases of diabetes worldwide (1, 2). Despite advances in pharmacological therapies, achieving sustained glycemic control remains challenging, highlighting the need for complementary strategies targeting upstream metabolic dysregulation. In recent years, the gut microbiome has emerged as a critical regulator of host metabolism, with accumulating evidence demonstrating its central role in the development and progression of T2D (3–5).

Alterations in gut microbial composition and function have been consistently observed across different stages of T2D, distinguishing affected individuals from non-diabetic controls (3). Mechanistically, the gut microbiome influences glucose homeostasis through multiple pathways, including modulation of insulin sensitivity, regulation of host inflammatory responses, and production of bioactive metabolites that interact with host metabolic signaling networks (6, 7). Notably, recent mechanistic studies have demonstrated that microbial carbohydrate metabolism and downstream metabolite production can directly contribute to insulin resistance and impaired glucose regulation. These findings position the gut microbiome and its metabolites as promising targets for nutritional and metabolic interventions in T2D.

Dietary strategies and nutrition-based interventions represent a practical and scalable approach to modulate the gut microbiome in metabolic diseases. Increasing evidence suggests that bioactive compounds derived from plant-based foods and herbal formulations can reshape microbial communities and alter metabolite profiles, thereby improving glycemic control. Several dietary-derived polysaccharides and phytochemicals have been shown to regulate glucose metabolism through microbiota-dependent mechanisms, linking nutritional intake to host metabolic outcomes (8–11). Moreover, components derived from Ophiopogon, Cornus, and Morus alba have demonstrated glucose-lowering and insulin-sensitizing effects in preclinical and clinical contexts, further supporting the concept that complex dietary herbal formulations may exert metabolic benefits through microbiome-mediated pathways (12–15).

QingYun7 (QY7) is a standardized dietary herbal formulation developed based on long-term clinical practice and composed of multiple plant-derived components with reported metabolic relevance. However, its glycemic efficacy and underlying microbiome-related mechanisms have not been systematically evaluated in a longitudinal clinical setting. In the present study, we investigated the metabolic effects of QY7 using an integrated preclinical and clinical framework. We first assessed its impact on glucose metabolism and gut microbiota in a diabetic rat model, and subsequently validated its glycemic efficacy in a prospective cohort of patients with T2D with repeated follow-up measurements. By combining longitudinal gut microbiome profiling, serum metabolomics, fecal microbiota transplantation (FMT), and mediation analysis, we aimed to elucidate a microbiota–metabolite–glucose regulatory axis underlying the metabolic effects of this dietary intervention.

Together, this study provides evidence supporting a nutrition-based, microbiota-targeted strategy for glycemic control in T2D and offers mechanistic insights into how gut microbiome modulation and metabolic reprogramming contribute to improved glucose homeostasis.

## Methods

### Preparation and identification of QY7

QY7 is an herbal-based dietary formulation composed of five plant-derived. ingredients, including *Rhizoma Atractylodis Macrocephatae* (baizhu, BZ), *Ophiopogon Japonicus (Linn. f.) Ker-Gawl* (maidong, MD), *Astragalus Membranaceus (Fisch.) Bunge* (huangqi, HQ), *Mori Folium* (sangye, SY), and *Cornus officinalis* (shanzhuyu, SZ), combined at a weight ratio of 1:1:1:3:2. All materials were purchased from Tongjuntang Pharmaceutical Co. Ltd. (Hangzhou, China) and authenticated by the Zhejiang Traditional Chinese Medicine Academy. Voucher specimen of the individual component was deposited at Zhejiang Traditional Chinese Medicine Academy. The chemical composition of QY7 has been characterized using Ultra-High-Performance Liquid Chromatography-Quadrupole Time-of-Flight Mass Spectrometry (UHPLC-Q/TOF-MS), as reported in previous study (16). For clinical use, two packets of QY7 (10g per packet) were soaked in warm water and consumed as daytime hydration across waking hours. For the animal experiment, the adult dosage was converted to rat-equivalent dosage based on body surface area, and adjust to three times for the rat experiment (i.e., 6.24 g/kg).

### Type 2 diabetes rat experiment

This animal experiment included three groups: the control group, which consisted of normal male Wistar rats, and two T2D model groups using male GK rats (Goto Kakizaki rats). Wistar rats are commonly used as controls for GK rats. The GK rats in the diabetes model group received vehicle, while rats in the treatment group received QY7 at a dose of 6.24 g/kg through the 16-week experiment. Each group contained six rats. At the end of the experiment, a glucose tolerance test (GTT) experiment was conducted. Serum, kidney, and liver samples were collected for biochemical analysis. All rats were provided by Laboratory Animal Center of Zhejiang Traditional Chinese Medicine Academy. All rats were housed in a 12-h light (7 a.m. to 7 p.m.) and 12-h dark (7 p.m. to 7 a.m.) cycle, with free access to water and food. The experiments were conducted under the Guidelines for Animal Experiment of Zhejiang Traditional Chinese Medicine Academe, and the protocol was approved by the animal ethics review committee of Zhejiang Traditional Chinese Medicine Academy (approval number of ethics protocol: 2022027).

### Study population and data collection

The human study was based on a prospective cohort and conducted at Tongde Hospital of Zhejiang Province, China (ClinicalTrial.org: NCT05312450). Patients were enrolled consecutively according to predefined criteria. Inclusion criteria were a clinical diagnosis of type 2 diabetes mellitus and age ≥ 18 years. Patients without T2D, malignant disease, pregnancy, or incomplete clinical data were excluded. These patients participated in a three-month anti-hyperglycemia program implemented in a local clinic, where all received QY7 combined with nutritional management. Patients took QY7 *via* drinking water across waking hours for three months. Dietary recommendations were provided based on each patient's food tolerance test and food glycemic index. All patients were instructed to avoid foods they could not tolerate and to choose fiber-rich foods with low glycemic index for their daily meals. As a standard care, a low-carbohydrate diet was recommended for all patients during the first week. The structure dietary guidance reflects real-world diabetes care rather than an experimental dietary manipulation, and QY7 was evaluated within this standard of care context. Throughout the treatment, the doses of patients' Western antidiabetic medications were gradually adjusted downward according to national guidelines for the prevention and control of diabetes in China (17). According to the records, 69 participants were newly diagnosed and had not received prior glucose lowing medication. Among the remaining 289 participants: for non-insulin agents, 74% achieved complete discontinuation (100% reduction), and 18% reduced doseage by more than 50%; for the insulin agent, 75.1% achieved complete discontinuation, and 16.3% reduced dosage by more than 50%. Thus, the majority of treated participants substantially discontinued both insulin and non-insulin agents while maintain improved glycemic profiles. Each patient received individualized followed up and detailed dietary guidance from a nutritionist. Patients self-monitored blood glucose using fingertip testing, including fasting, 2-h post-prandial, and random glucose, and reported to their nutritionist. Blood glucose data were collected at baseline (V1), the first week, the first month (V2), the second month (V3), and the third month of therapy (V4), as well as the first and second week after the therapy. Blood and stool sample were collected at baseline (V1), the first month (V2), the second month (V3), and the third month of therapy (V4).

### 16 S rDNA sequencing

In the rat experiment, intestinal contents were collected from three sites: small intestine, cecum, and colorectum. Fecal DNA was isolated using the Qiagen QIAamp DNA Stool Mini Kit (Qiagen, Düsseldorf, Germany), and analyzed by 16S rDNA sequencing on the Illumina platform (Illumina Inc., CA, USA) based on published methods (18), in which the V3–V4 region was amplified and sequenced. QIIME 2 was used to analyze the sequence reads.

### Metagenome analysis

In the human study, there were 406 stool samples, consisting of 159 samples at baseline (V1), 109 samples at the first month of treatment (V2), 78 samples at the second month of treatment (V3), and 60 samples at the third month of treatment (V4).

Fecal samples from participants were collected during on-site study visits. Before DNA extraction, the samples were kept frozen at −80°C. 0.5g of stool material was used to extract total genomic DNA with the Fast Pure Stool DNA Isolation Kit (Magnetic bead) (MJYH, Shanghai, China) according to manufacturer's instructions. The concentration and purity of extracted DNA were determined using Synergy HTX and NanoDrop2000, respectively. DNA quality was assessed on a 1% agarose gel. The extracted DNA was fragmented to an average size of approximately 400 bp using Covaris M220 (Gene Company Limited, China) for paired-end library construction. The library was constructed using the NEXTFLEX Rapid DNA-Seq kit (Bioo Scientific, Austin, TX, USA). Paired-end sequencing was performed on an Illumina NovaSeq™ X Plus (Illumina Inc., San Diego, CA, USA) at Majorbio Bio-Pharm Technology Co., Ltd. (Shanghai, China) using the NovaSeq X Series 25B Reagent Kit according to the manufacturer's instructions.

Next, raw sequencing reads were quality-controlled using PRINSEQ. Taxonomic profiling of the shotgun metagenomic data was performed using MetaPhlAn4 (19), which utilizes a library of clade-specific markers to provide pan-microbial quantification at the species level. MetaPhlAn4 was run with default settings, and only species-level relative abundance data were considered in this study.

### Serum metabolomic analysis

There were 384 serum samples used for metabolomic profiling, including 154 samples at baseline (V1), 101 at the first month of treatment (V2), 76 at the second month of treatment (V3), and 53 at the third month of treatment (V4), along with 40 quality control (QC) samples. Pooled QC samples were included in the sample queue to assess system stability and repeatability, and were processed alongside the biological samples.

Metabolomic profiling was performed using UHPLC-QTRAP-MS. Briefly, serum samples were extracted using methanol/acetonitrile/water, with stable-isotope internal standards added. The serum samples were then shaken, incubated on ice, and centrifuged. The supernatant was filtered, dried, and redissolved, and then injected into UHPLC (1,290 Infinity LC, Agilent Technologies) coupled to a QTRAP MS (6,500+, Sciex). HILIC and C18 columns were used for separation, with gradient elution. Analysis was performed in positive/negative modes using MRM for metabolite quantification. Detailed information regarding sample preparation and detection is provided in the Supplementary material.

### FMT animal experiment

Male C57BL/6J mice aged 5 weeks were provided by the Laboratory Animal Center of Westlake University (Hangzhou, China). All mice were housed in a 12-h light (7 a.m. to 7 p.m.) and 12-h dark (7 p.m. to 7 a.m.) cycle, with free access to food. Mice were divided into four groups: control group, antibiotics group (ABx group), T2D patient group (before treatment, BT group), and T2D patients with QY7 therapy group (after treatment, AT group). Each group contained 9–10 mice. The ABx, BT, and AT groups received a two-week antibiotic cocktail treatment consisting of 0.5 g/L vancomycin, 1 g/L ampicillin, 1 g/L neomycin, and 1 g/L metronidazole, all administered via drinking water *ad libitum* (20). After two weeks, mice in ABx group returned to normal water till the end. Mice in the BT and AT groups underwent a recovery period with regular water for at least half a day before FMT. For FMT, there are three human donors, each providing stool samples at two time points (before and after QY7 therapy), resulting in a total of six donor samples. Each donor's sample was transplanted to 2–3 recipient mice. Human fecal samples were stored at −80°C. At least 200 mg of frozen stool was resuspended in 10 times volumes of sterilized PBS. Two sterilized magnetic beads were added, and the mixture was vortexed for 3 min and allowed to settle by gravity for 15 min on ice. A total of 300 μL of the supernatant was transplanted into recipient mice (21–24). Mice were maintained on normal chow diet and water till the end. A glucose tolerance test was performed two and five weeks after FMT. Serum insulin levels were measured at five weeks after FMT. The experiments were conducted under the Guidelines for Animal Experiment of Westlake University, and the protocol was approved by the institutional Animal Ethics Committee.

### RT-PCR analysis

RT-PCR was used to assess bacterial load in mouse fecal samples. Total DNA was extracted from mouse fecal samples using TIANamp stool DNA kit (#DP328-02, TIANGEN). Real-time qPCR was performed using SYBR Green (A25777, Thermo Fisher Scientific, USA) and the CFX connect Real-Time System. The raw bacterial content was assessed based on the number of cycles of the 16s primer at the same DNA concentration. The sequences of 16S primers were in the V3–V4 conservative region, 338Forward: 5′-ACTCCTACGGGAGGCAGCA-3′, 806Reverse: 5′-GGACTACHVGGGTWTCTAAT-3′.

### Statistical analysis

Animal experimental data shown in this study were expressed as the mean ± standard error of the mean (SEM) unless otherwise specified. Differences in gut bacteria between groups were evaluated using the Mann–Whitney *U-*test. Other comparisons between two groups were conducted using a two-tailed Student's *t*-test. A *P*-value < 0.05 was considered statistically significant.

Characteristics of the patients with T2D were presented as mean [standard deviation (SD)] for continuous variables and as frequency (percentage) for categorical variables. Blood glucose levels (e.g., fasting glucose, 2-h post-prandial glucose, and random glucose) measured at the first week, the first month, the second month, and the third month of therapy, and first and second week after therapy were compared with baseline using paired Wilcoxon signed-rank test. For subgroup analysis, the above analyses were stratified by age [older (age>median age) and younger (age < =median age)], gender, and BMI [overweight/obesity (BMI≥24 kg/m^2^), (25) and normal weight (BMI < 24 kg/m^2^)], respectively.

For metagenomics data, species with a prevalence greater than 10% were included in the following analyses. A total of 106, 76, and 57 paired samples between V2 and V1, V3 and V1, and V4 and V1, respectively, were available. α diversity analysis based on paired samples was performed using paired Wilcoxon test. Principal coordinates analysis (PCoA) based on Bray-Curtis distance was conducted using gut microbiota data available at all four timepoints (*N* = 39), and association between timepoints and β-diversity dissimilarity was assessed using permutational ANOVA (PERMANOVA). Differential species between the paired samples (V2 vs. V1, V3 vs. V1, and V4 vs. V1) were identified using paired Wilcoxon test, with a false discovery rate (FDR) < 0.25 considered statistically significant. The Benjamini-Hochberg procedure was applied to control FDR. Associations between differential species and blood glucose were evaluated by mixed linear model, adjusted by sex, age, BMI, duration of T2D months, and timepoint.

For metabolite analysis, 204 metabolites were included with a coefficient of variation (CV) in QC samples < 30% and a prevalence > 80% in at least one timepoint. Missing values were filled with half of the minimum value. Paired Wilcoxon test was used to select differential metabolites between V2, V3, and V4 vs. V1, based on 96, 74, and 51 paired samples, respectively. Metabolites with FDR < 0.25 across all three comparisons (V2 vs. V1, V3 vs. V1, and V4 vs. V1) were selected as perturbated metabolites during QY7 therapy. Utilizing metabolomics data available at all four timepoints (*N* = 31), PCoA analysis was performed based on Bray-Curtis distance, and examined the association between timepoints and β-diversity dissimilarity using PERMANOVA. Linear trend test was performed using linear mixed-effect model, with visiting time treating as a continuous variable, adjusted for age, gender, BMI, and T2D duration. Linear mixed-effect model was performed to examine the associations between the identified gut bacteria and perturbated metabolites, adjust for sex, age, BMI, duration of T2D months, and timepoint.

Mediation analysis (using the *mediate* function from the R package *mediation*) was conducted to evaluate the mediating role of blood metabolites in linking microbial species and blood glucose. The mediation analysis was carried out in two parts. In the first part, the mediation analysis was performed using samples available across all four timepoints (29 samples) with complete metagenomic and metabolomic data. This analysis focus on shared differential bacteria (47 species) and shared differential metabolites (67 metabolites) identified at V2, V3, and V4 relative to V1 (Supplementary Figure S5d). In this mediation analysis, linear mixed-effect model was first used to select bacteria significantly associated with blood glucose, and then select metabolites significantly associated with the blood glucose-related species. Subsequently, the mediation analysis was conducted to determine whether the selected metabolites could mediate the association between the selected species and blood glucose. In the second part, the mediation analysis was performed using repeated-measured samples from V2 and V1 (93 samples), V3 and V1 (73 samples), and V4 and V1 (48 samples), respectively, following the same methods described above (Supplementary Figure S7). Differential bacteria and metabolites selected in V2, V3, and V4 compared with V1 (paired Wilcoxon test, FDR < 0.25) were included in the corresponding mediation analysis. All analyses were performed in R (version: 4.4.1).

## Results

### Metabolic and gut microbiome responses to QY7 in diabetic rat

**Figure 1:**
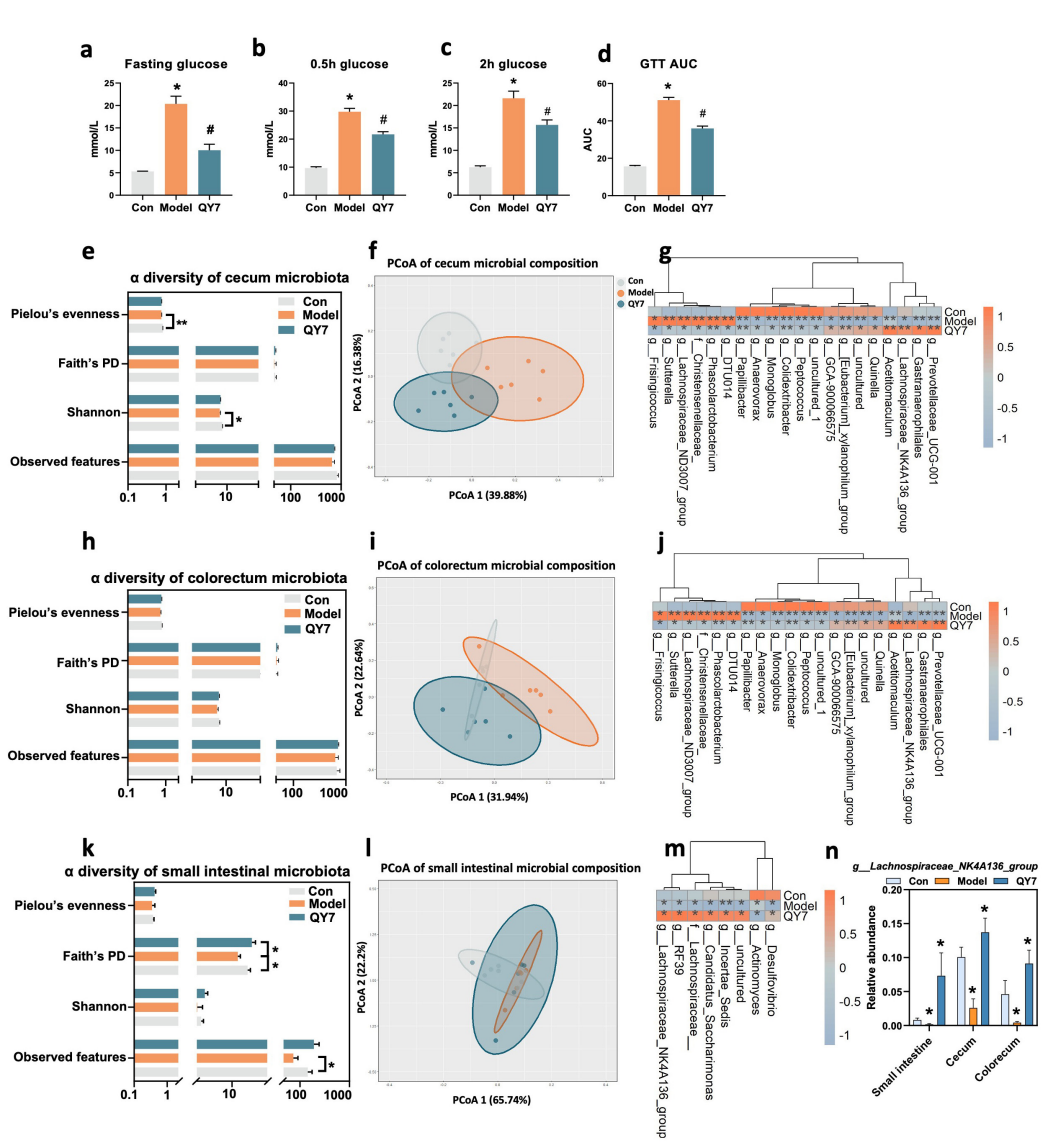
QY7 therapy could maintain glucose homeostasis in diabetic rats accompany with the remodeling of gut microbiome. **(a–d)**, Fasting glucose, 0.5h glucose, 2h glucose, GTT AUC. Data are mean ± SEM, *n* = 6, *p < 0.05 corresponding with Con group, #*p* < 0.05 corresponding with Model group. *P* values were calculated using two-tailed Student's *t*-tests. **(e, h, k)**, α diversity of small intestinal, cecum, and colorectum microbiota. For biochemical indicators, *p < 0.05 **p < 0.01, *p* values were calculated using two-tailed Student's *t*-test. **(f, i, l)**, Microbial composition by PCoA analysis of small intestinal, cecum, and colorectum. **(g, j, m)**, All changed genus which relative abundance was significantly changed in T2D model and then regulated by QY7 in small intestinal, cecum, and colorectum. **(n)**, Relative abundance of *g__Lachnospiraceae_NK4A136_group* which was the only genus that significantly changed in model group and reversed by QY7 in *three* intestinal parts simultaneously. For gut microbiome, *p < 0.05 **p < 0.01, *p* values were calculated using Mann-Whitney *U*-test, and the model group was compared with con group, QY7 group was compared with model group. Data are mean ± SEM, *n* = 6.

The metabolic effects of QY7 were first evaluated in a diabetic rat model using a daily dietary intervention of 6 g/kg for 16 weeks. Compared with the model group, fasting glucose, 0.5 h glucose, 2 h glucose, and the area under the curve of the glucose tolerance test (GTT AUC) were markedly elevated but were significantly reversed following QY7 intervention (Figures 1a–d). In addition to glycemic improvement, QY7 intervention attenuated metabolic disturbances, including normalization of elevated HbA1c, total glycated hemoglobin (GH), serum high-density lipoprotein (HDL), maintenance of renal urea levels, and reduction of the liver injury marker aspartate aminotransferase (AST) (Supplementary Figures S1a–j). Together, these results indicate that QY7 confers broad metabolic benefits in diabetic rats.

We next examined whether glucose improvements were accompanied by alterations in the gut microbiome. QY7 intervention significantly increased α-diversity of the small intestinal microbiota and shifted β-diversity of the cecal and colorectal microbiota toward that of control animals (Figures 1e, f, h, i, k, l). At the genus level, eight genera altered in the model group were reversed by QY7 in the small intestine (Figure 1g), while twenty genera showed consistent restoration in the cecum and colorectum (Figures 1j, m). Notably, *g__Lachnospiraceae_NK4A136_group* was consistently enriched following QY7 intervention across all three intestinal segments (Figure 1n). This genus has been reported as a beneficial microbial taxon associated with improved glucose (26–28). These findings suggest that dietary intervention with QY7 not only improves glycemic and metabolic profiles in diabetic rats but also restores gut microbial composition.

### Ameliorated fasting, random, and postprandial blood glucose were observed in patients with T2D after QY7 therapy

**Figure 2:**
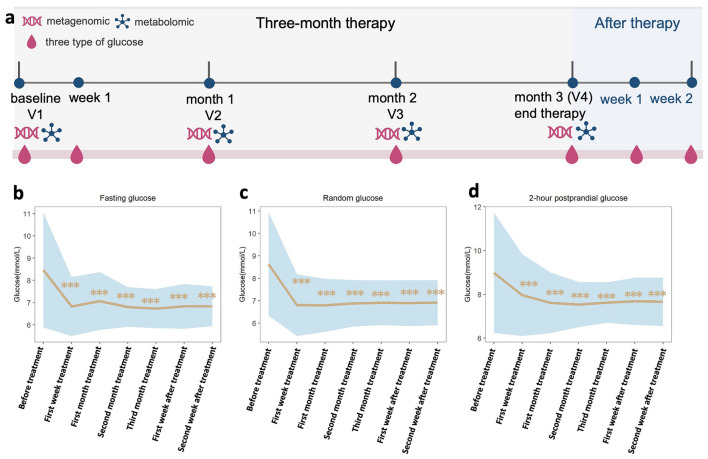
Blood glucose in T2D patients were ameliorated during therapy. **(a)** clinical flowchart. **(b–d)**, fasting glucose, random glucose, and 2-h postprandial glucose were detected at seven timepoints, before treatment, first week treatment, first month treatment, second month treatment, third month treatment, first week after treatment, and second week after treatment. Wilcox single rank test shown the significant reduce of blood glucose after treatment compared with before treatment in paired samples. *** present *p* value < 0.001.

Among 358 enrolled patients with T2D receiving a comprehensive anti-hyperglycemia program incorporating QY7 for three months in a prospective cohort (Figure 2a, Supplementary Figure S2), the mean (SD) age at baseline was 57.9 (9.4) years (50.8% female) (Table 1). Blood glucose levels were significantly decreased after the first week of treatment, with mean (SD) levels decreased from 8.46 (2.57) to 6.83 (1.33), 8.62 (2.30) to 6.80 (1.38), and 8.98 (2.74) to 7.96 (1.87) for fasting glucose, random glucose, and 2-h postprandial glucose, respectively (all *P* < 0.001; Figures 2b–d; Supplementary Figure S3), and remained stable for two weeks after treatment. No clinically significant abnormalities were observed in the safety assessments throughout the treatment period (Supplementary Table S1).

**Table 1:** The characteristics of study patients with type 2 diabetes at baseline.

Characteristics	Study patients (*N* = 358)
Age, years, mean (SD)	57.9 (9.4)
Female, *n* (%)	182 (50.8)
BMI, kg/m^2^, mean (SD)	24.8(3.8)
Overweight/obesity, *n* (%)^*^	187 (52.2)
Duration of T2D, months, mean (SD)	92.7 (76.5)
Duration of treatment, days, mean (SD)	104.2(30.7)

^*^Defined by BMI ≥ 24 kg/m^2^.

### Gut microbiome in patients with T2D was significantly restored after QY7 therapy

**Figure 3:**
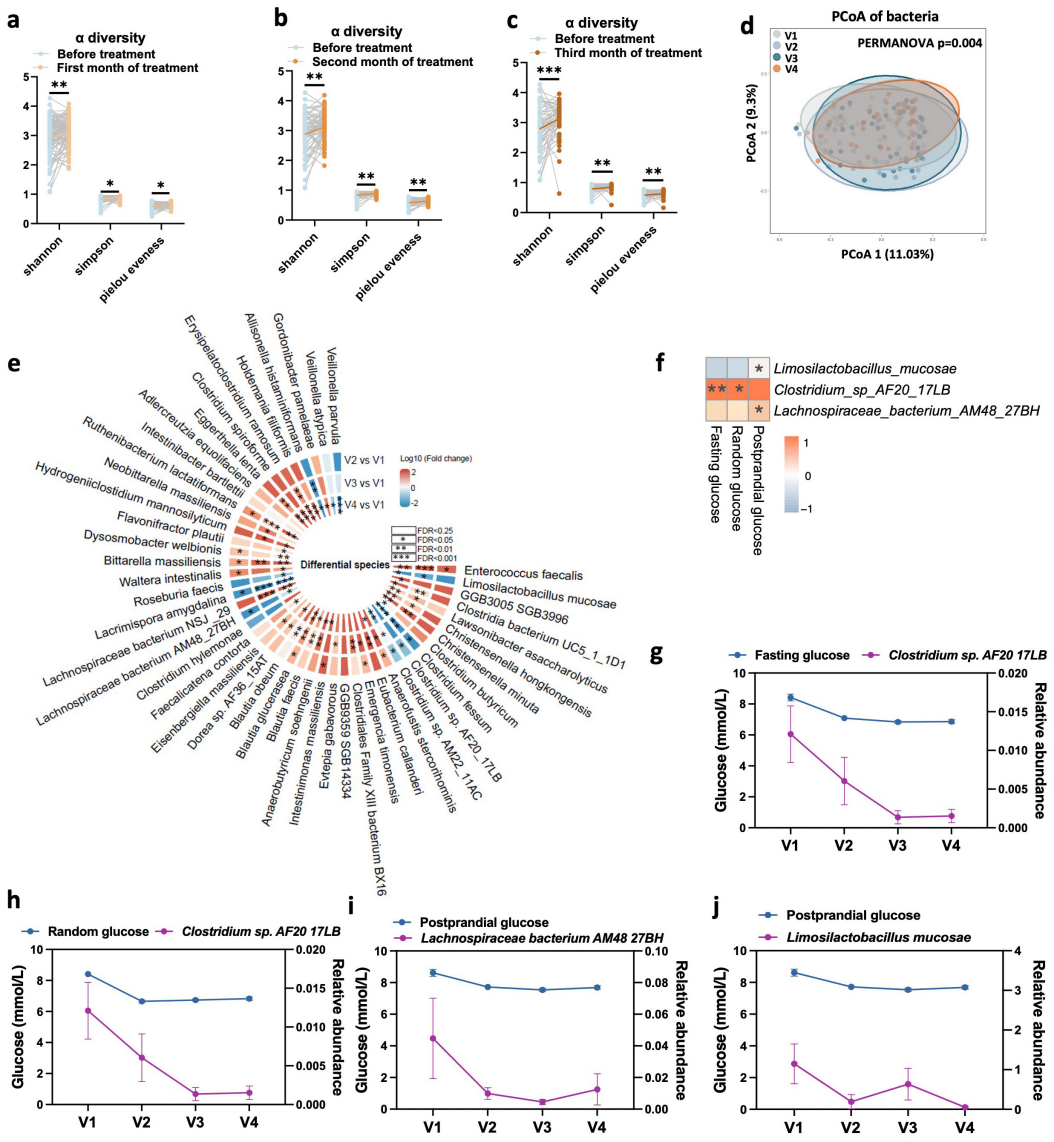
Gut microbiome was significantly changed and associated with glucose during whole therapy in T2D patients. **(a–c)**, line graph shown the significant increase of three α diversity indicators after treatment (first month of treatment: V2, second month of treatment: V3, third month of treatment: V4) compared with baseline (before treatment: V1) in paired samples. Wilcox single rank test, *, **, *** present *p* value < 0.05, < 0.01, and < 0.001. **(d)**, PCoA analysis shown the significantly change of gut microbiome structure in therapy period based on metagenome data of 39 full present participants. The *p*-value detected by PERMANOVA is 0.004. **(e)**, significantly changed species at V2, V3, and V4 compared with V1 separately. Wilcox single rank test, the p value after FDR of all presented species was less than 0.25, *, **, *** present FDR < 0.05, < 0.01, and < 0.001. **(f)**, mix linear model shows the characterize bacteria, which was significantly associated with glucose, in whole therapy process. Wilcox single rank test, the *p* value after FDR of all species was less than 0.25, *, **, *** present FDR < 0.05, < 0.01, and < 0.001. **(g–j)**, Expression trend of characterize bacteria during whole therapy.

Gut microbiota α diversity indices were significantly increased when comparing the first month (V2), the second month (V3), and the third month of therapy (V4) with the baseline (V1), respectively (species-level Shannon, Simpson, and Pielou evenness: *P*_V2vs.V1_ = 4.6x10^−3^, 0.013, and 0.015; *P*_V3vs.V1_ = 1.1x10^−3^, 3.5x10^−3^, and 4.8x10^−3^; *P*_V4vs.V1_ = 3.7x10^−4^, 1.09x10^−3^, and 2.7x10^−3^; Figures 3a–c). PCoA analysis based on Bray-Curtis distance at the species level showed a significant change in bacterial structure after therapy (PERMANOVA *P* = 0.004, Figure 3d). Compared with V1, the abundances of 70, 118, and 107 species were dramatically reversed at V2, V3, and V4, respectively, with 47 species overlapping between V2 vs. V1, V3 vs. V1, and V4 vs. V1 (FDR < 0.25; Figure 3e). These longitudinal microbial changes mirrored those observed in the diabetic rat model, supporting a consistent association between glycemic improvement and restoration of gut microbiota composition following QY7 therapy.

### QY7-modulated gut microbiome reduces blood glucose

To determine whether the beneficial effects of QY7 were rely on the restored gut microbiota, association analysis between restored gut bacteria and blood glucose in patients, and FMT experiment were performed.

In the association analysis, three species, *Limosilactobacillus mucosae, Clostridium sp. AF2017LB* and *Lachnospiraceae bacterium AM4827BH* were significantly associated with fasting glucose, random glucose, and 2-h postprandial glucose among whole therapy with mixed linear analysis model (FDR < 0.25; Figures 3f–j). These associations were also significant at each timepoint across separate analysis (Supplementary Figures S4a–c). Besides, there were 16, 7, and 13 species shown a significant association with blood glucose at V2, V3, and V4 across separate analysis (Supplementary Figures S4a–c).

**Figure 4:**
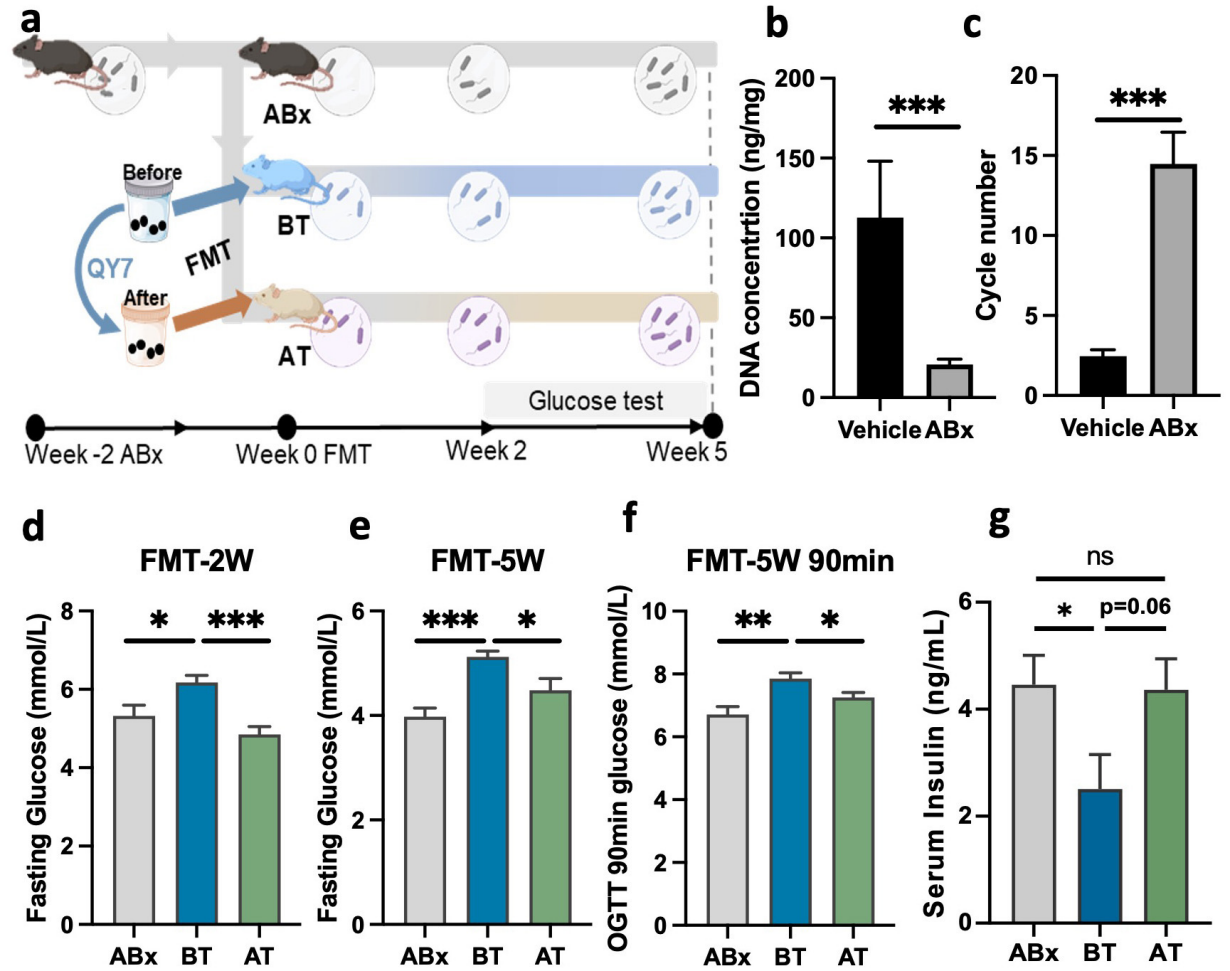
FMT experiment confirmed that the QY7 remodeled gut microbiome could improves blood glucose. **(a)**, Experimental flow chart. **(b)**, DNA concentration of stool sample after two weeks antibiotic intervention, *n* = 4–6. **(c)**, 16 s cycle number of stool sample DNA by qPCR analysis, *n* = 4–6. **(d)**, Fasting glucose in three group after two weeks FMT, *n* = 9–10. **(e, f)**, Fasting glucose, and 90 min glucose in OGTT after five weeks FMT, *n* = 6–9. **(g)**, Fasting serum insulin level at the end point, *n* = 5–6. Data are mean ± SEM, *p < 0.05 **p < 0.01, *p* values were calculated using two-tailed Student's *t*-test.

In FMT experiment, two weeks of cocktail antibiotics successfully removed the vast majority of intestinal bacteria from the mice (Figures 4a–c), then followed by FMT. Mice receiving fecal sample collected before QY7 treatment (BT group) shown a significantly elevated blood glucose levels (*P* < 0.05; Figure 4d); in contrast, mice transplanted with fecal samples after QY7 treatment (AT group) showed a significant reduction in blood glucose compared with the BT group, and the levels of blood glucose were comparable between the AT group and the ABX group (Figure 4d). After five weeks post-FMT, mice in the BT group showed a significant increase in blood glucose at 0 min and 90 min, while blood glucose levels were significantly reduced in the AT group compared with the BT group (Figures 4e, f; Supplementary Figure S4d). In addition, serum insulin levels in the BT group were significantly reduced compared with those in the ABx group, whereas the levels in the AT group were restored and comparable to those in the ABx group (Figure 4g). These findings confirmed the essential role of gut microbiota in QY7's glycemic effect, and informed our subsequent investigation into the functional implications of the altered gut microbe, with a focus on microbiota-associated serum metabolites.

### Serum metabolites in patients with T2D show substantial changes in response to QY7 therapy

**Figure 5:**
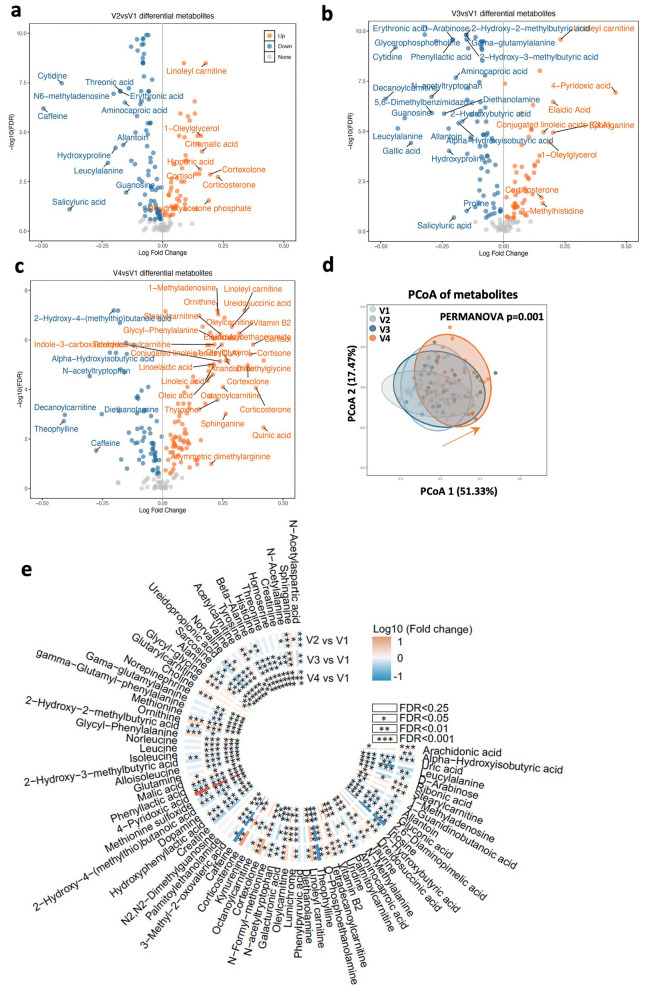
Serum metabolomics in T2D patients was significantly changed during therapy. **(a–c)**, V-plot graph shown the significant changed metabolites at three timepoints after therapy. Blue dots indicate significantly reduced metabolites; orange dots indicate significantly increased metabolites. Wilcox single rank test, the *p* value after FDR less than 0.25 was considered as significant. Metabolites with name present |LogFC| > 0.15. **(d)**, PCoA analysis shown the significantly change of serum metabolites structure in therapy period based on metabonomic data of 31 full present participants. The *p*-value detected by PERMANOVA is 0.001. **(e)**, significantly changed metabolites at three timepoints after therapy compared with V1 separately. Wilcox single rank test, the *p* value after FDR of all species was less than 0.25, *, **, *** present FDR < 0.05, < 0.01, and < 0.001.

To further understand the potential mechanisms by which the gut microbiota could drive host pathophysiology, we hypothesized that metabolites are an important class of molecules that are involved in the QY7-microbe-glucose interaction. After technical criteria, 478 targeted metabolites were included in the following analysis. Then, we screened differential metabolites with the criteria of paired samples and FDR < 0.25 in each timepoint. Compared with V1, a total of 161, 164, and 166 metabolites had changed significantly at V2, V3, and V4 respectively, which shown in Figures 5a–c. PCoA analysis at metabolite level shown the significantly change of metabolite structure after start therapy (Bray-Curtis distance, PERMANOVA *P* = 0.001, Figure 5d). By profiling the serum metabolites, 106 metabolites were changed among three timepoints (FDR < 0.25), Figure 5e shown 67 metabolites with FDR less than 0.05 (all data shown in Supplementary Table S2). We also found that 39 of the 106 differential metabolites belonged to class amino acids, peptides, and analogs, and the results of the pathway enrichment analysis showed that QY7 treatment significantly affected multiple metabolic pathways including valine, leucine and isoleucine biosynthesis pathway, arginine biosynthesis pathway, and arginine and proline metabolism pathway (Supplementary Figures S5a, b). These results suggest that the alterations in serum metabolic profiles were synchronized with the gut microbiome, which tentatively validate our hypothesis. Next, we will explore the mediating effects of serum metabolites directly with mediation analysis.

### Serum metabolites mediated the robust gut microbe-blood glucose association in participants

To comprehensively identify key metabolites involved in mediating the contribution of the gut microbiota to the glucose-lowering effects of QY7, we conducted mediation analyses from two complementary perspectives. First, a longitudinal analysis was performed by integrating differential metabolites consistently observed at V2, V3, and V4 with differentially species through correlation and mediation analyses, Supplementary Figure S6a shown the analysis flowchart. Second, cross-validation analyses were conducted independently at each time point, in which time point-specific differential metabolites were correlated with differential species and further evaluated using mediation analysis, Supplementary Figures S6b–d shown the analysis flowchart.

**Figure 6:**
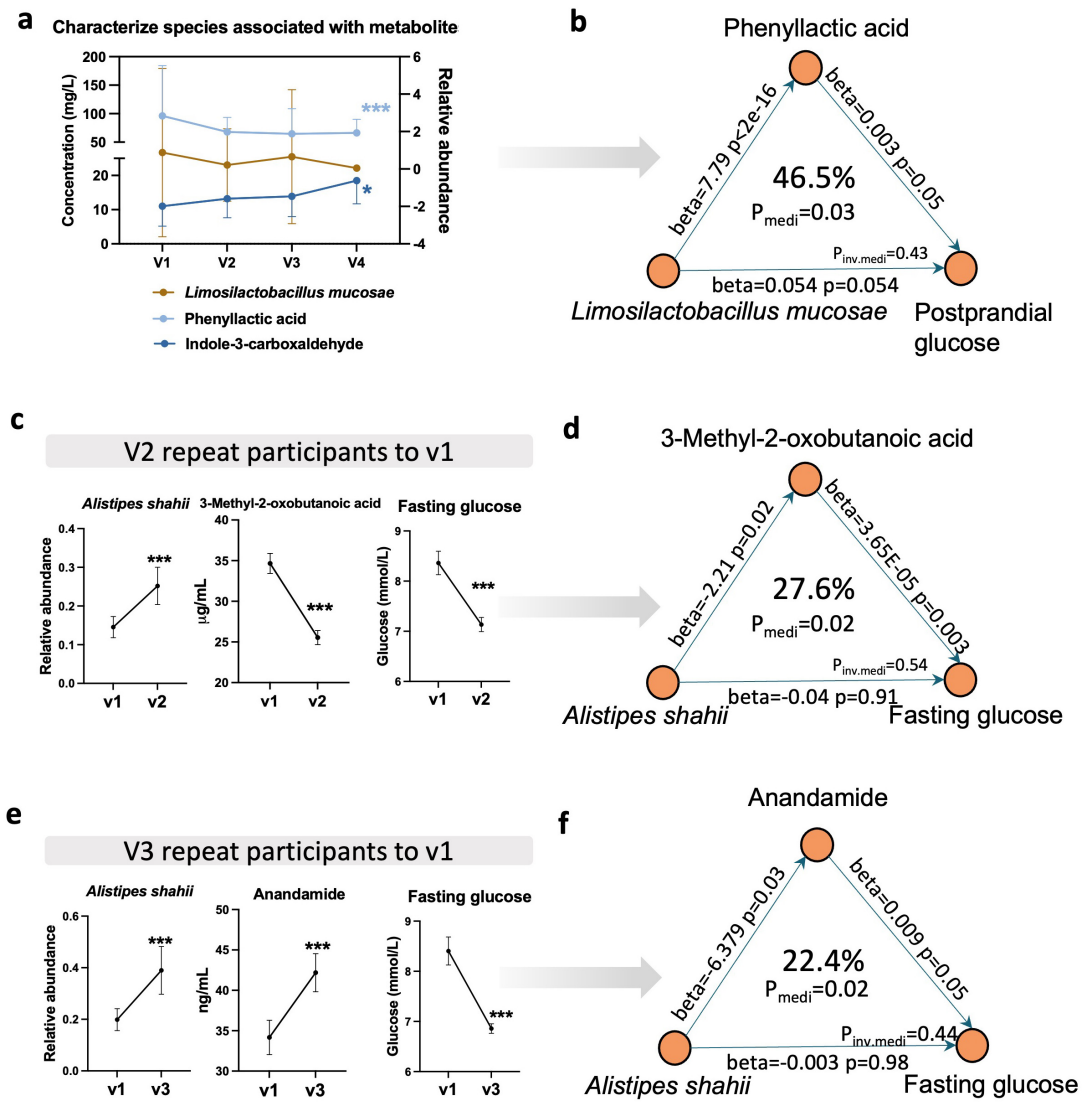
Serum metabolites mediate blood glucose-gut microbial interaction. **(a)**, characterize bacteria *Limosilactobacillus mucosae* associated with Phenyllactic acid and Indole-3-carboxaldehyde, with estimate 0.027 and 0.10, respectively. Wilcox single rank test, the *p* value after FDR of all species was less than 0.25, *, *** present FDR < 0.05, and < 0.001. **(b)**, the *Limosilactobacillus mucosae* causally contributed to postprandial glucose through Phenyllactic acid (Pmediation = 0.03, 46.5%) during therapy. **(c, d)**, the *Alistipes shahii* causally contributed to fasting glucose through 3-Methyl-2-oxobutanoic acid (Pmediation = 0.02, 27.6%) at time v2. Line graph shows the expression changes of three factors in v1(before treatment) and v2 (first test after treatment) repeat participants (*n* = 93). **(e, f)**, the *Alistipes shahii* keep contributed to fasting glucose through anandamide (Pmediation = 0.02, 22.4%) at time v3. Line graph shows the expression changes of three factors in v1(before treatment) and v3 (second test after treatment) repeat participants (*n* = 73). Test was performed with paired Wilcoxon rank test, present mean ± SEM, *** present *p* < 0.001. medi, mediation effect; inv.medi, inverse mediation effect. Data used for analysis from same timepoint.

In the longitudinal analysis, 67 metabolites (Figure 5e, differential species across V2, V3, and V4) were involved in association analysis with three blood glucose-associated species (Figure 3f, *Limosilactobacillus mucosae, Clostridium sp. AF2017LB* and *Lachnospiraceae bacterium AM4827BH*), results shown that phenyllactic acid and indole-3-carboxaldehyde were significantly associated with *Limosilactobacillus mucosae* (FDR < 0.25, Figure 6a). Based on this, further mediation analysis showed that phenyllactic acid mediated the association between *Limosilactobacillus mucosae* and postprandial glucose (Figure 6b, P_medi_ = 0.03, 46.5%).

In the cross-sectional analysis, the association of differential bacteria and differential metabolites, and following mediation analysis were performed in V2, V3, and V4 separately (FDR_mediation_ < 0.05; Supplementary Figures S6b–d, Supplementary Table S3). Notably, the mediation effects of serum metabolites on the *Alistipes shahii*-fasting glucose association were replicated at timepoint V2 and timepoint V3 (Figures 6c–f). Mediation analysis suggested that *Alistipes shahii* could negatively regulate fasting glucose by decreasing 3-Methyl-2-oxobutanoic acid level at first month and decreasing Anandamide level at second month.

Collectively, these findings indicate coordinated alterations in gut microbiota and serum metabolites, with mediation analysis supporting a potential role of circulating metabolites in linking microbial shifts to glycemic parameters.

## Discussion

In this study, we demonstrate that QY7, implemented as a diet-based intervention, associated with improved glycemia in T2D, supported by consistent evidence from both a diabetic rat model and a prospective clinical cohort. The observed decreases in fasting (−1.63 mmol/L), random (−1.82 mmol/L), and postprandial glucose (−1.02 mmol/L) indicated meaningful regulation and reduced glycemic variability in patients with T2D. Importantly, mechanistic validation in FMT mice and further mechanistic exploring in mediation analysis provided support for a microbiota-mediated contribution to glycemic regulation. These findings suggest that QY7 exerts a rapid and sustained effect on multiple dimensions of glycemic control.

Diet-based and botanical interventions have long been explored as complementary strategies for metabolic disorders, yet clinical evidence and mechanistic understanding remain limited according to insufficiently powered studies and heterogeneous intervention designs (29–36). To address this gap, we conducted a prospective cohort study integrating QY7 with standardized nutritional management and guideline-based adjustment of conventional antidiabetic medications (37). This integrated approach reflects real-world clinical practice for diabetes management, where dietary modulation and medication optimization are routinely combined. Given the single-arm design of the clinical cohort, causal inference regarding independent clinical efficacy should be interpreted cautiously. The structured nutritional management program included an initial short-term low-carbohydrate phase, which may have contributed to early glucose reductions. However, sustained glycemic trends were observed beyond the initial phase, indicates that QY7-induced alterations in the gut microbiota exert an independent and sustained metabolic regulatory effect. Notably, substantial pharmacological de-escalation occurred during the intervention: among previously treated participants, the majority reduced or discontinued both insulin and non-insulin glucose-lowering agents while maintaining improved glycemic indices. Although these findings cannot exclude residual confounding, QY7 was associated with substantial and sustained glycemic improvement, providing clinically relevant evidence that complements existing preclinical findings and underscores the value of rigorous clinical evaluation for diet-based interventions.

Previous studies have reported that certain botanical or formula-based dietary interventions could improve glycemic outcomes through modulation of the gut microbiome and microbial metabolites (38). Systematic reviews further support that such interventions, when combined with standard care, can influence gut microbial structure and metabolic outcomes (39, 40). Compared with these studies, our work extends existing evidence by integrating a large prospective cohort with repeated sampling, animal validation, fecal microbiota transplantation, and multi-omics analyses. This muti-layered design strengthens biological plausibility by linking clinical associations with experimentally transferable metabolic phenotypes.

Consistent alterations in gut microbial diversity were observed across intestinal segments in the rat model and longitudinally in patients, indicating a systemic modulation of the gut ecosystem. Although certain taxa enriched in rats, such as *g__Lachnospiraceae_NK4A136_group*, were not directly annotated in human metagenomic data, this discrepancy likely reflects interspecies differences in microbiome composition and sequencing resolution. Importantly, the FMT experiments demonstrated that microbiota shaped during QY7 intervention could transmit glycemic effects to recipient mice, providing causal evidence that gut microbiota alterations contribute to metabolic improvement. Given the central role of insulin in glucose homeostasis (41), our findings further suggest that microbiota-driven mechanisms may influence glycemic control, potentially through modulation of insulin dynamics, although this requires further experimental validation.

In the FMT model, metabolic improvement was most pronounced at two weeks and showed partial attenuation by week five, a pattern consistent with early donor microbiota engraftment followed by ecological stabilization in recipients. The single-dose FMT design was intended to test causal transmissibility rather than long-term maintenance. In the meantime, the FMT experiment partially isolates microbiota-driven effects from concurrent dietary or medication influences. Strategies such as repeated transplantation or approaches that enhance microbial persistence may be required to sustain long-term metabolic effects.

Among the identified microbiota-associated metabolites, phenyllactic acid emerged as a key mediator. Phenyllactic acid is a microbial metabolite produced by lactic acid bacteria, including Lactobacillus-related species, via aromatic amino acid metabolism. The observed association between *Limosilactobacillus mucosae* and phenyllactic acid is therefore biologically plausible and supports a mechanism whereby diet-induced microbial shifts influence host glucose regulation through specific microbial metabolites.

Beyond serving as essential nutrients, BCAAs function as signaling molecules regulating glucose and lipid metabolism through pathways such as PI3K/AKT/mTOR (42). Their significant alteration, together with identified mediation linkages between gut bacteria, serum metabolites, and blood glucose, suggests that BCAA-related pathways may represent another key mechanism linking dietary modulation, microbial metabolism, and glycemic regulation.

Several limitations should be acknowledged. First, the absence of a randomized control arm limits definitive attribution of glycemic changes to QY7 independent of dietary or behavioral modification. Second, although all participants completed longitudinal glucose monitoring, multi-omics sample availability decreased over time due to voluntary non-compliance with repeated biological sampling, more repeated sample could increase the statistical power for later time-point analyses. Third, mediation analyses support potential causal pathways, but direct experimental validation of specific metabolites and signaling targets is needed to further substantiate these mechanisms.

## Conclusions

In conclusion, this study demonstrates that QY7, applied as a dietary intervention, is associated with meaningful improvements in glycemic control in T2D. Through integration of animal experiments, cohort study, fecal microbiota transplantation, longitudinal multi-omics analyses, and mediator analysis, we provide evidence that gut microbiota alterations and downstream serum metabolites contribute to these effects. Specific microbial taxa, including *Limosilactobacillus mucosae* and *Alistipes shahii*, and metabolites such as phenyllactic acid, 3-methyl-2-oxobutanoic acid, and anandamide, emerge as potential functional mediators linking dietary modulation to glucose regulation. Together, these findings highlight the translational evidence of targeting the gut microbiome–metabolite axis through dietary strategies for T2D management, and provide a basis for future randomized controlled trals to confirm its therapeutic efficacy.

## Data Availability

The data presented in this study are publicly available. The data can be found here: https://www.ncbi.nlm.nih.gov/sra, accession PRJNA1275584.
