# Supplementary material for: Microbiota-metabolites interaction associated with glycemic improvement following a dietary herbal intervention in type 2 diabetes
Source: Front Nutr. 2026 Apr 13;13:1793130. doi: 10.3389/fnut.2026.1793130 (PMC13111312; doi:10.3389/fnut.2026.1793130)
Supplement: Supplementary file 4 [file Data_Sheet_1.pdf]

Supplementary figures

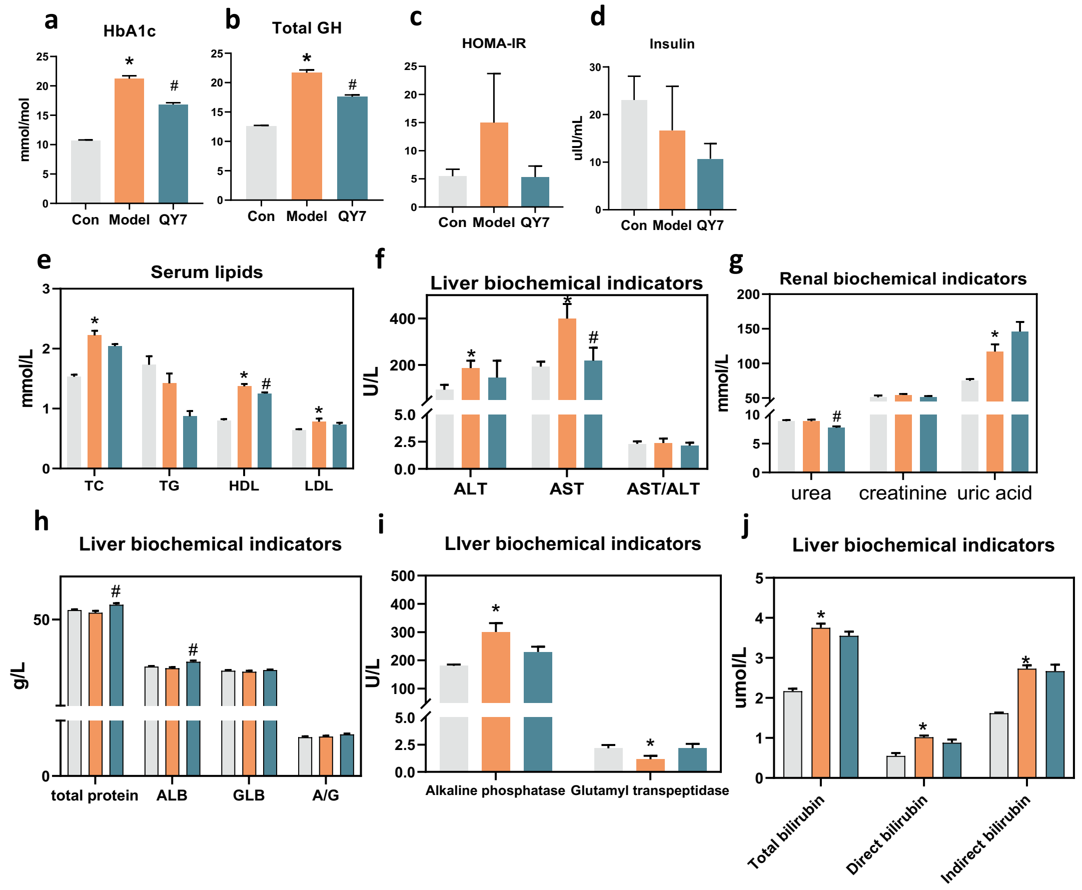

eFigure1 QY7 therapies ameliorate infect on biochemical indicators. a-b, HbA1c (glycosylated hemoglobin), total GH. c-d, insulin related indicators: HOMA-IR, and serum insulin; e, serum lipid-related biochemical indicators; f, liver biochemical indicators.; g, renal related indicators: urea, creatinine, and uric acid; h-j, liver biochemical indicators: total protein, serum albumin(ALB), serum globulin(GLB) , the ratio of ALB to GLB (A/G), alkaline phosphatase, glutamyl transpeptidase, total bilirubin, direct bilirubin, and indirect bilirubin. *$p<0.05$ #$p<0.05$, p values were calculated using Mann-Whitney U-test, and the model group was compared with con group, QY7 group was compared with model group. Data are mean ±SEM, n=6.

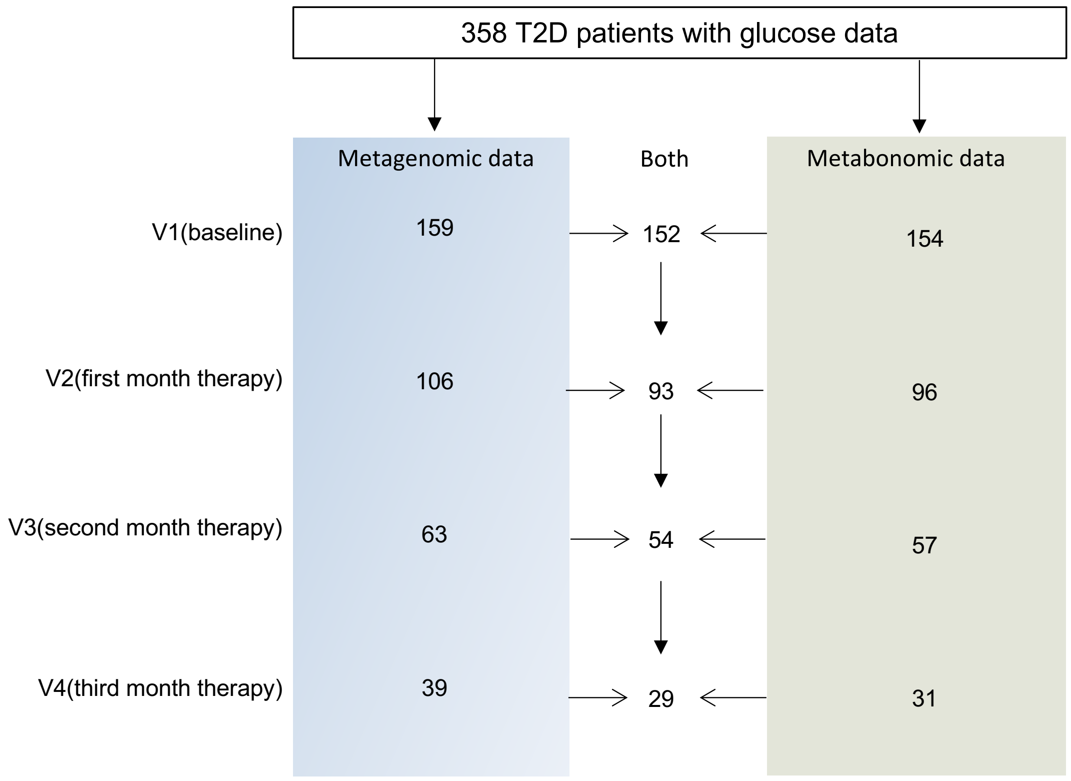

eFigure2 Flow chart of QY7 therapy's clinical trial.

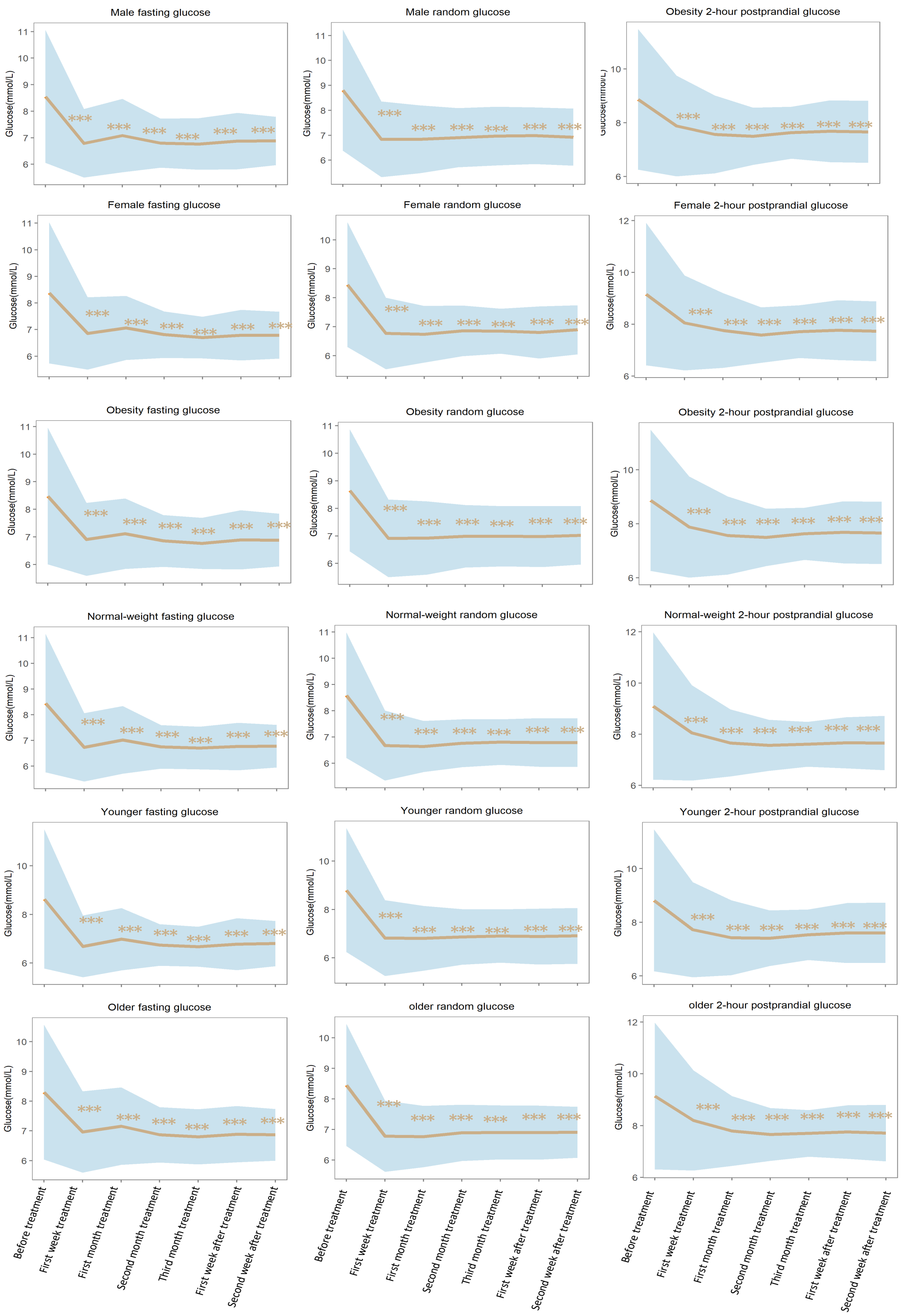

eFigure3 The glycemic regulation effect of therapy was not affected by gender, age, and BMI. Subgroup analysis was used to test the stability of blood glucose homeostasis maintained by therapy. Sex was divided into male and female; obesity and non-obesity were defined by whether the BMI≦ 24, participants were categorized as old and young according to whether they are older than the median age. Wilcox single rank test shown the significant reduce of blood glucose after treatment compared with before treatment in paired samples at six timepoints. *** present p value <0.001.

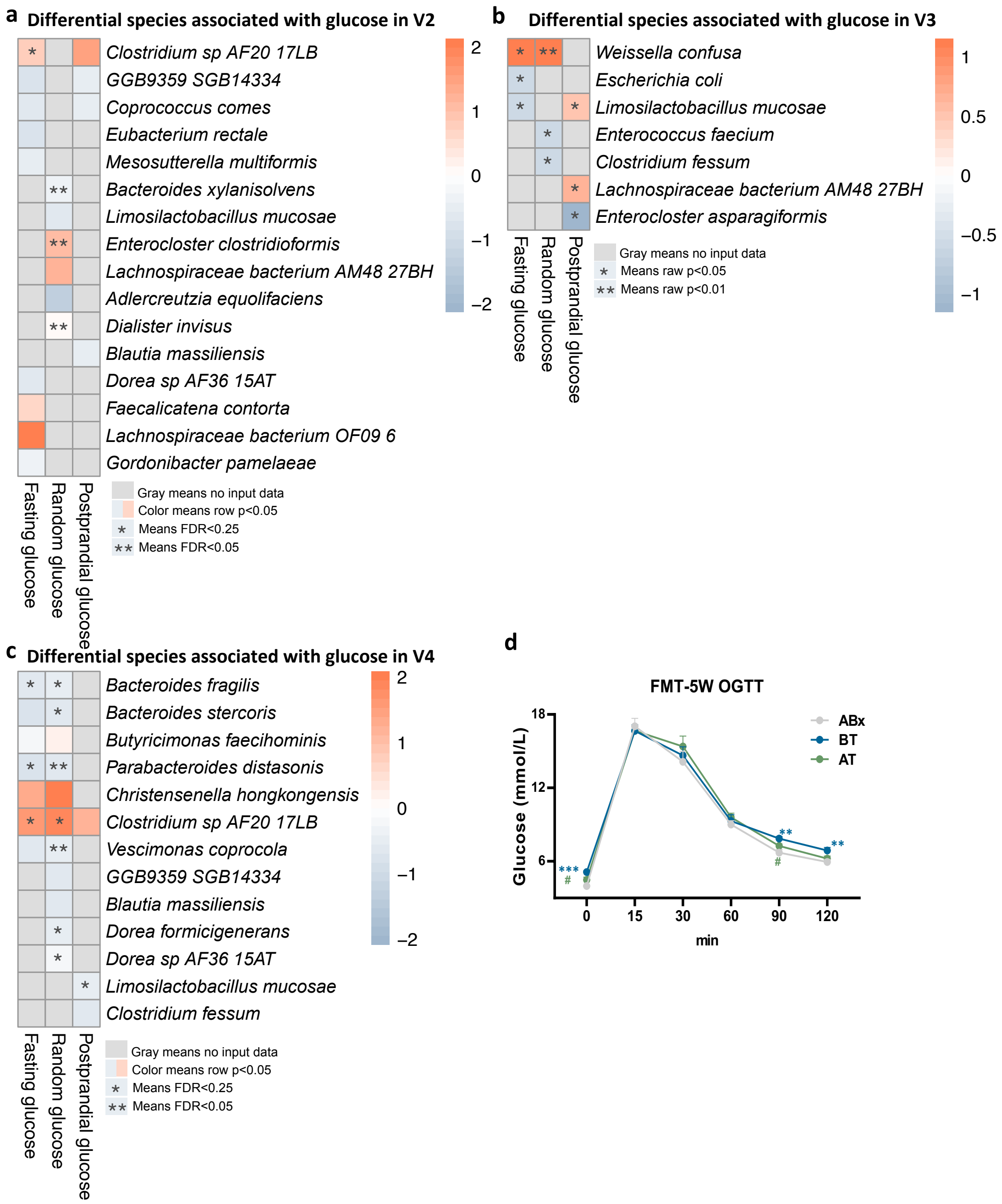

eFigure4 a-c the characterize bacteria which was significantly associated with glucose in each timepoint. Heatmap shown the differential bacteria and glucose association in each timepoint according to mix linear model. d the OGTT after 5 weeks fecal microbiome transplantation. Data are mean ±SEM, *p<0.05 **p<0.01 means BT group compared with ABx group, # p<0.05 ## p<0.01 means AT group compared with BT group, p values were calculated using two-tailed Student's t-tests.

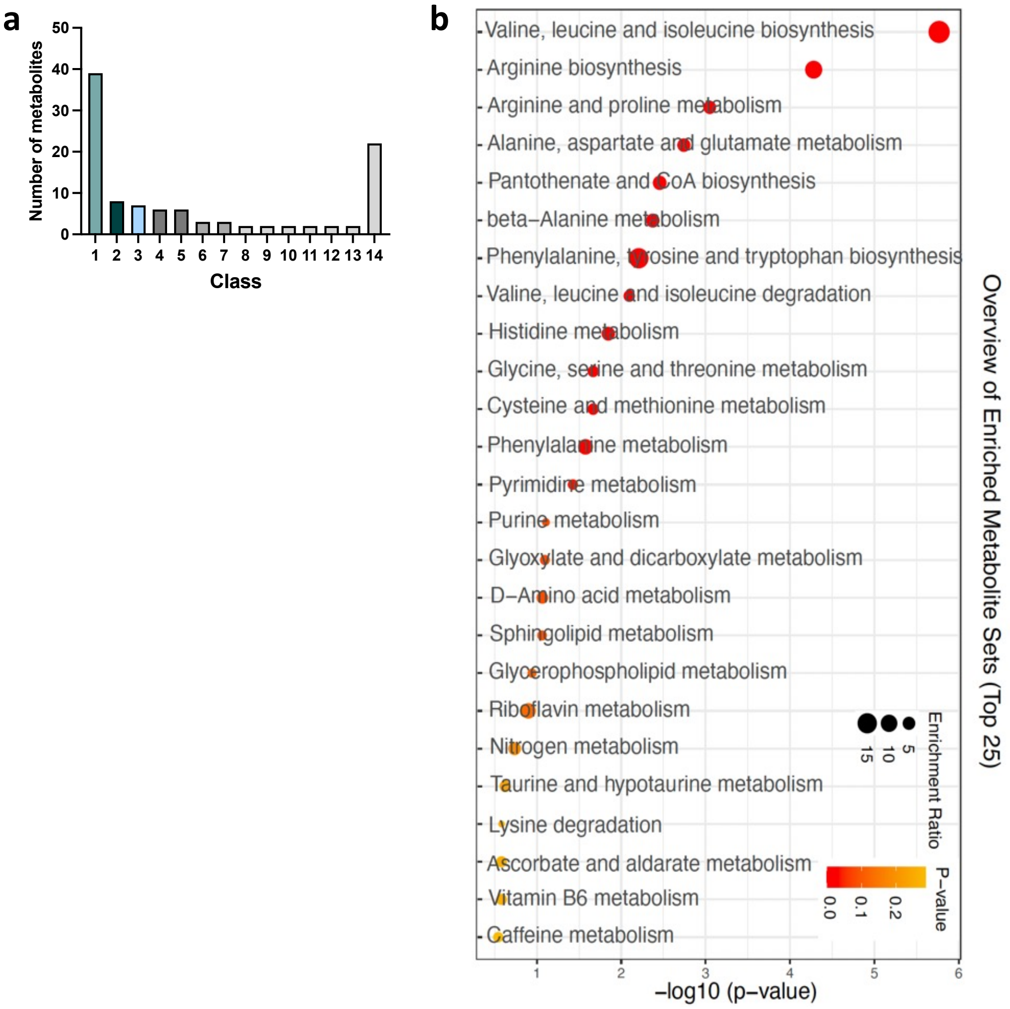

eFigure5 Serum metabolomics in T2D patients was significantly changed after therapy. a, significantly changed metabolites at three timepoints after therapy compared with V1 separately. Wilcox single rank test, the p value after FDR of all species was less than 0.25, *, **, *** present FDR <0.05, <0.01, and <0.001. a, Class of 106 differential metabolites.1.Amino acids, peptides, and analogues; 2.Fatty acid esters; 3.Fatty acids and conjugates; 4.Amines; 5.Carbohydrates and carbohydrate conjugates; 6.Hydroxysteroids; 7.Purines and purine derivatives; 8.5'-deoxy-5'-thionucleosides; 9.Alloxazines and isoalloxazines; 10.Alpha hydroxy acids and derivatives; 11.Benzoic acids and derivatives; 12.Phenylpropanoic acids; 13.Short-chain keto acids and derivatives; 14.other class. b, Enriched pathways based on 106 differential metabolites via MetaboAnalyst(https://www.metaboanalyst.ca).

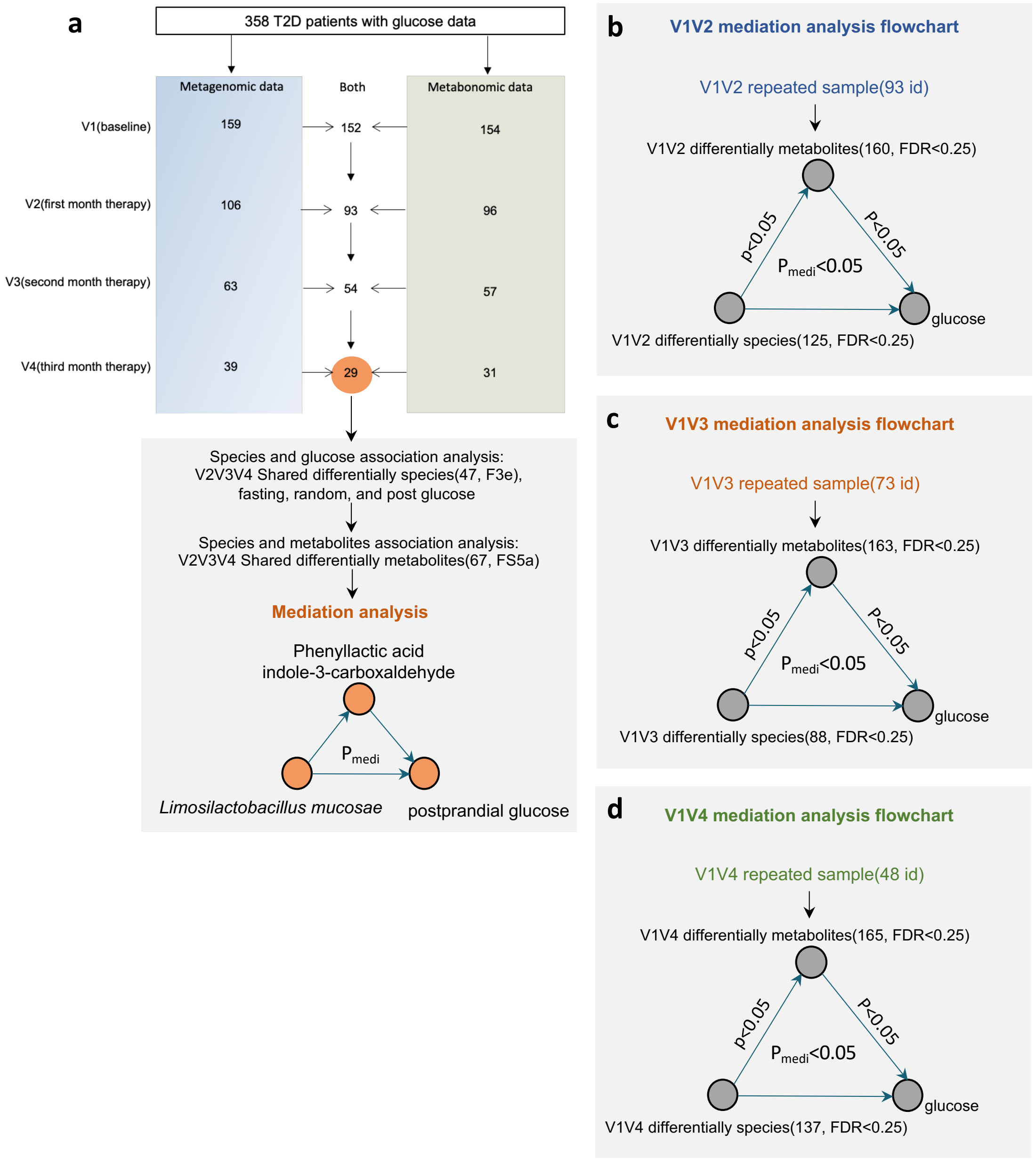

eFigure6 a, the analysis flowchart of mediation analysis based on longitudinal data; b-d, the analysis flowchart of mediation analysis based on cross-sectional data.
